# The rationale for the treatment of long-Covid symptoms – A cardiologist's view

**DOI:** 10.3389/fcvm.2022.992686

**Published:** 2022-09-15

**Authors:** Elisabeth Schieffer, Bernhard Schieffer

**Affiliations:** Department of Cardiology, Angiology and Critical Care Medicine, University Hospital Marburg (UKGM), Philipps University Marburg, Marburg, Germany

**Keywords:** lipid – hybrid nanoparticles, HDL - cholesterol, long-Covid syndrome, histamin challenge, cholesterol, renin-angiotensin system, kalkrein-kinin system

## Abstract

The ongoing coronavirus disease 2019 pandemic left us with thousands of patients suffering from neurological, cardiovascular, and psychiatric disorders named post-acute sequelae of COVID-19 or just *long-Covid*. In parallel, the vaccination campaigns against SARS-CoV-2 spike protein saved millions of lives worldwide but long-Covid symptoms also appeared rarely following vaccination with a strong overlap to the “canonical” long-Covid symptoms. A therapeutic strategy targeting both, post-VAC and post-SARS-CoV-2 long-Covid symptoms is warranted since exposure to the S-protein either by vaccination or SARS-CoV-2 infection may trigger identical immuno-inflammatory cascades resulting in long-Covid symptoms.

## Introduction

Our vaccination campaign at Marburg University Hospital started on Christmas 2020 to mitigate the regional impact of the COVID-19 pandemic. We thereby aimed to protect our high-risk patients namely elderly and immune-deficient patients as well as healthcare colleagues. In parallel, we were forced to treat an increasing number of patients suffering from long-term neurological, pneumatological, and cardiac symptoms following either severe or moderate SARS-CoV-2 infection called *long-Covid* or post-acute sequelae of COVID-19 (PASC). We established an interdisciplinary outpatient clinic in response to this increasing number of patients. From autumn 2021 onward, several patients appeared with typical long-Covid symptoms who never suffered from SARS-CoV-2 infections. We realized that these symptoms developed days to weeks after vaccination regardless of which vaccine was used (only BioNTech Pfizer, Moderna, and AstraZeneca were used at that time in Germany) and were changing over time (personal observation of more than 350 post-vaccination patients). Interestingly, the clinical presentation of these so-called post-vaccination (post-VAC) patients showed a strong symptom-overlap with long-Covid patients in terms of chest pain, fatigue, shortness of breath, and a variety of neurological disorders i.e., headache, pain, paresthesia, insomnia, and polyneuropathy. In addition, post-VAC patients often suffered from additional dermatological and gastro-intestinal disorders i.e., diarrhea, skin rush, and burning skin indicating that “hyper-allergic/hyperinflammatory affection” might also be involved. However, not all patients following SARS-CoV-2 infection and even significantly lesser patients post-VAC developed PASC so it is of outstanding interest to understand the underlying pathophysiology.

So far, it was the canonical understanding that using the sequence of an antigen, namely the SARS-CoV-2 spike protein for vaccination, might trigger the same pathways, but- under particular circumstances or an immunological environment- can result in the development of identical symptoms named long-Covid. Moreover, if this latter is true, are these pathways suitable targets for therapeutic interventions, and is post-VAC with its defined starting point, the clear onset of symptoms, and immunological responses a potential model to understand the pathophysiology of COVID-19? While writing this review, the manuscript by Trougakos et al. (1) was published discussing the spike protein hypothesis and potential pathways involved in adverse effects after COVID-19 vaccines. This molecular mimicry of immuno-inflammatory cascades resulting in symptoms ranging from shortness of breath, chest pain, migraine, headache, fever, and “brain fog” to other neurological symptoms which should be the target of future therapeutical interventions.

## Two sides of the same evil - SARS-CoV-2 and its vaccines

SARS-CoV-2 has infected hundreds of millions of people and accounts for millions of death worldwide (2). In an unprecedented effort, vaccines were developed to protect from SARS-CoV-2 infection and broadly administered (3). Different vaccine platforms are nowadays available which all have in common that they use SARS-CoV-2 spike protein as antigen. In clinical trials and post-marketing evaluations, vaccines showed efficiency against infection and severe disease with acceptable side effects (4–6). With increasing numbers of vaccines being applied, increasing numbers of serious adverse events (SAE) were reported (7, 8) and shared by people on social media due to the lack of national registries in most countries such as Germany. Clinical symptoms vary from mild to severe - as seen in long-Covid patients and can develop weeks after vaccination, making it even more challenging for physicians and patients. Even though there has been enormous progress in the understanding of COVID-19 infection, the knowledge about PASC is limited and even more for the post-VAC syndrome (PVS). A plethora of medical and sociodemographic risk factors for long-Covid is known including gender (female sex), older age, obesity, asthma, and others (9, 10) but it remains discouraging that so far (august 2022) no evidence regarding the prediction for the development of either long-Covid or PVS exists.

**Figure 1:**
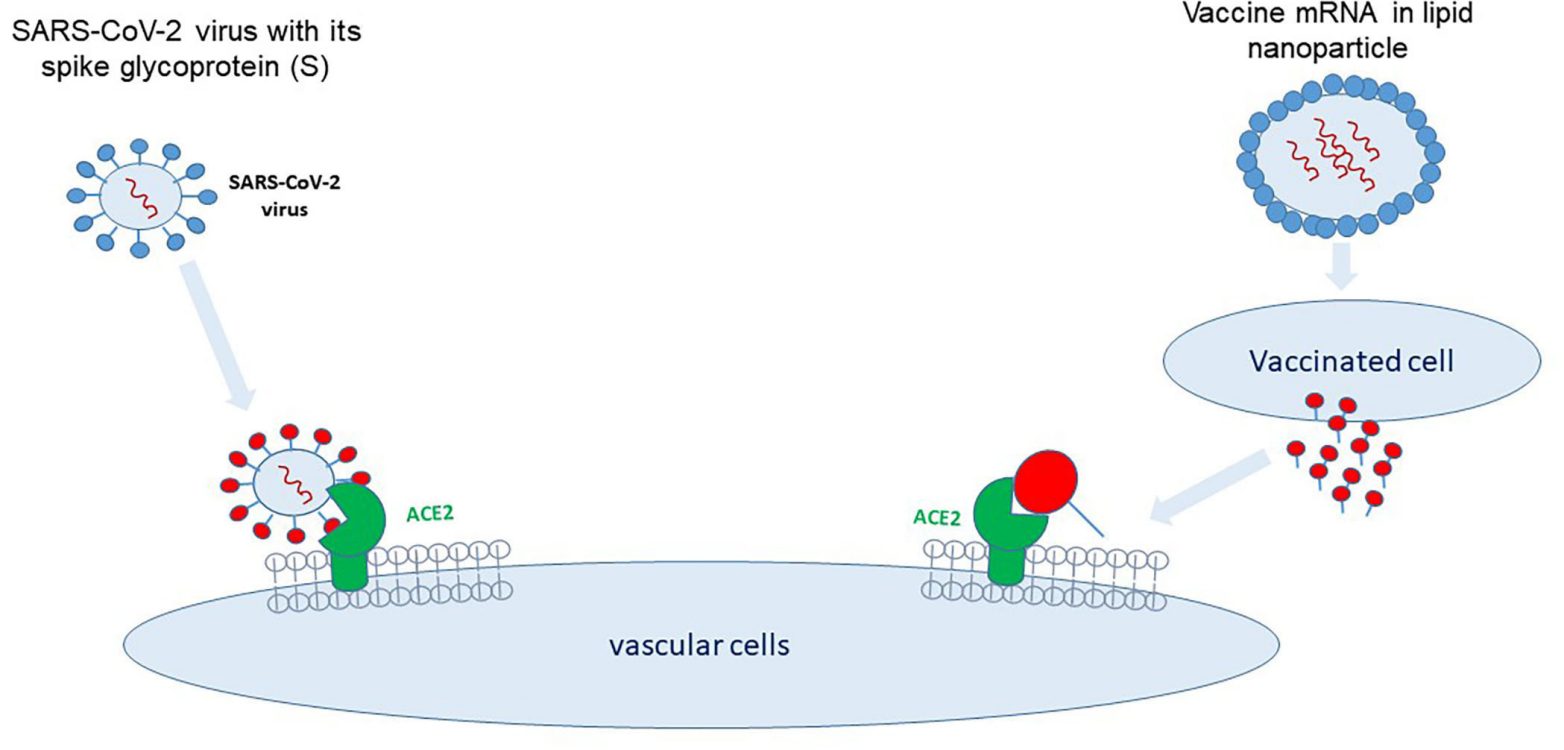
Two sides of the same evil. SARS-CoV-2 binds via its spike protein to the angiotensin-converting enzyme (ACE)2-receptor located on multiple cell types, i.e., vascular endothelial cells, immune cells, and pulmonary epithelial cells. mRNA vaccines encode for the SARS-CoV-2 spike protein which is synthesized and released by the transfected cells and binds to cells carrying the ACE2-receptor.

However, several hypotheses need to be discussed concerning the underlying pathophysiology:

*The spike-hypothesis*. The spike in S1-antigen has been detected as early as 1 day after vaccination in the plasma of 11 from 13 healthcare workers (11). The spike protein may induce endothelial dysfunction and a leakage of the blood-brain barrier (12). In addition, binding of the vaccines-spike protein to angiotensin-converting-enzyme 2 (ACE2) could promote an alternate pathway, inducing renin-angiotensin-system (RAS) imbalance and inflammation (1). Thus, spike protein binding/fusion with the ACE2-receptor might be the initial step leading to long-Covid symptoms following infection or vaccination (two sides of the same evil, Figure 1).

*The anti-idiotype antibody hypothesis*. Due to molecular mimicry, the involvement of anti-idiotype antibodies has been discussed (13). In this regard antibodies against the S-protein-induced antibodies might be generated and potentially bind to the original antigen which is the ACE2 receptor as well (13). This latter may occur uncontrolled following SARS-CoV-2 infection as well as in post-VAC patients (13), thereby unbalancing the RAS and blocking anti-inflammatory pathways.

*The antibody-complex-hypothesis*. Alternatively, Junqueira et al. (14) showed that the Fcγ receptors on (LPS-pre-stimulated) blood monocytes were able to recognize the SARS-CoV-2-antibody complex, resulting in an activated inflammasome (14). In this regard, Okuya et al. found antibody-enhancement antibodies in 63% of samples from acute or recovered COVID-19 patients and this was Fcγ receptor and complement (C1q) mediated which might account for increased infectivity in some patients (15).

*The autoantibody-hypothesis*. Another mechanism could be due to autoantibodies (AAB) that have been detected in acute COVID-19 (16) as well as in PASC (17). Binding of AAB to the ACE2-receptor could potentially result in ACE2 internalization, ACE2 shedding, and subsequently an imbalance within the RAS. Papola et al. detected antibodies to angiotensin II type 1 receptor (AT1Rab) during SARS-CoV-2 infection and patients with more severe disease had less AT1Rab than patients with moderate disease, suggesting a protective role of AT1Rab in acute infection (18). In patients with PASC functional AAB with activating or neutralizing activities against G-protein coupled receptors like adrenoceptors, AT1R, muscarinic M2- and the endothelin-receptor have been described (17). The pattern of AAB can differ individually and it remains to be elucidated whether they are an epiphenomenon. In addition, what's the impact of AAB on the pathophysiology and subsequently on clinical symptoms in PASC and post-VAC?

*The vaccine-adjuvant-hypothesis*. Reactions against the adjuvant were discussed previously (19). Nevertheless this is unlikely to explain the overlap in clinical symptoms seen in PASC and post-VAC just with the exposure to the vaccine-adjuvant.

Finally, patients with PASC can present with a variety of symptoms including fatigue, dyspnea, sleep disorder, and concentration difficulties (20). These symptoms were reported from post-VAC patients as well. As described above, the convincing hypothesis regarding the underlying mechanisms includes an imbalance within the RAS and subsequently a disturbed immune reaction. Therefore, it is tempting to speculate that - at least in part - identical underlying pathophysiological mechanisms develop PVS. Taken together, it is our understanding that PASC and PVS share identical immuno-inflammatory pathways and therefore post-VAC can serve as a model to analyze the pathophysiology of long-Covid.

### Role of the RAS-bradykinin-axis

**Figure 2:**
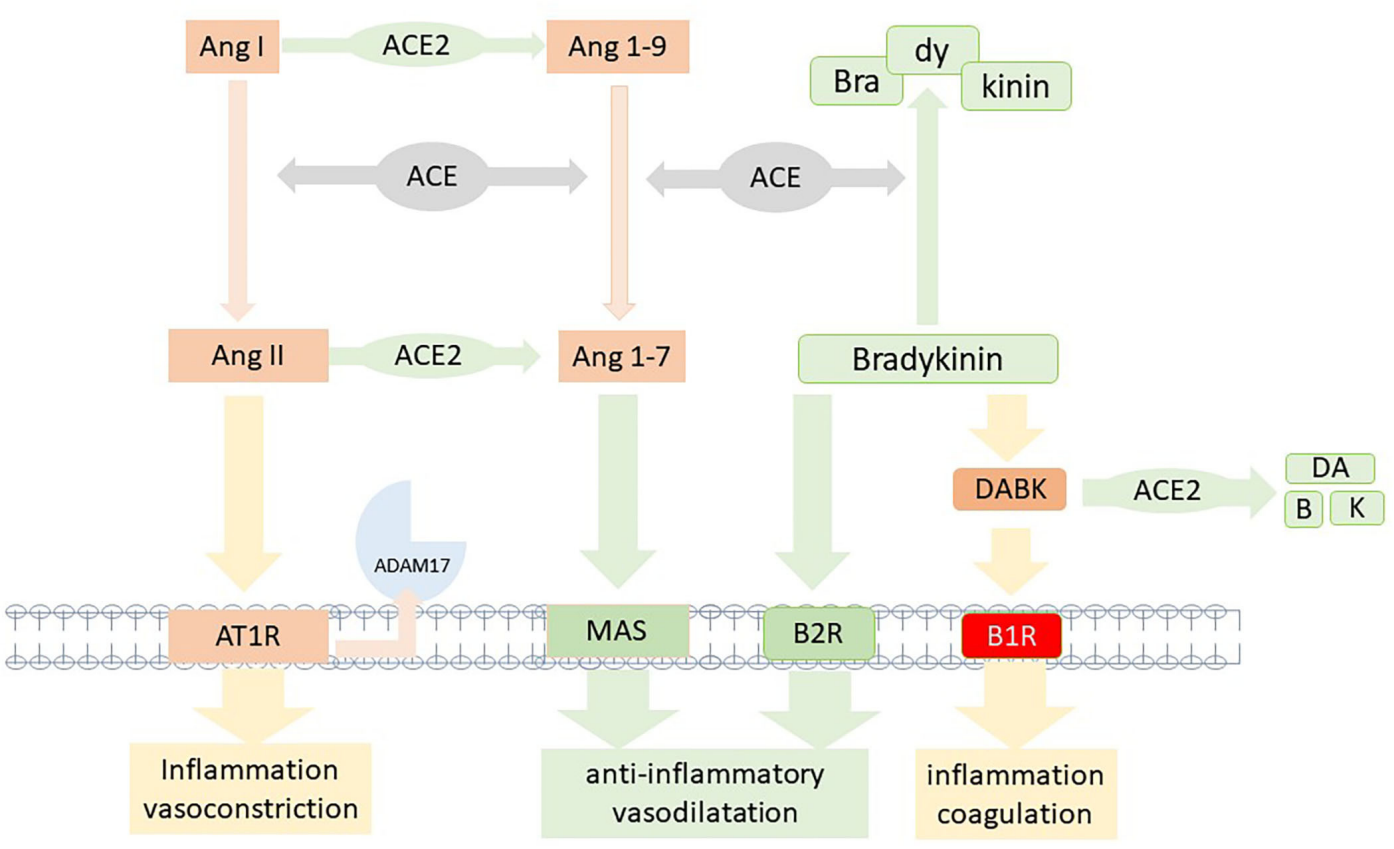
Model of the two competing axis of the renin-angiotensin system. In brief, AngiotensinII/Angiotensin II type 1 receptor (AngII/AT1R) and the Ang (1-7)/MAS receptor; the counterbalancing bradykinin system with degradation due to angiotensin-converting enzyme (ACE) and [des-Arg9]-bradykinin (DABK) by angiotensin-converting enzyme 2 (ACE2). Inactivation of ACE2 due to infection or vaccine would impair the anti-inflammatory Ang (1-7)/MAS and DABK degrading pathways, resulting in RAS imbalance and bradykinin-1 receptor (B1R) activation.

The renin-angiotensin system (RAS) elicits its biological effects via two signaling pathways summarized in Figure 2. The canonical angiotensin-I-converting enzyme1 (ACE1), angiotensin II (Ang II), angiotensin II receptor type 1 (AT1R) axis (ACE1/AngII/AT1R) inducing inflammation, fibrotic remodeling, and vasoconstriction. The counteracting pathway via angiotensin-I-converting enzyme 2 (ACE2) transforming angiotensin (1-7) [Ang (1-7)] which binds to the Mas-receptor [ACE2/Ang-(1-7)/MAS pathway] and promotes antifibrotic, anti-inflammatory pathways and vasodilatation (21). Imbalance of these two systems has been linked to developing diseases like lung injury, hypertension, and heart failure (22, 23).

Upon binding of spike protein to ACE2, membrane-bound ACE2 is decreased due to either internalization or shedding of the ACE2-spike protein complex by ADAM17. This mechanism results in the downregulation of the anti-inflammatory ACE2/ANG-(1-7)/Mas-AT2R pathway (21). Thus, spike protein-ACE2 binding leads to an imbalance within the RAS system by shifting it to the proinflammatory ACE/ANGII/AT1R axis and the subsequent release of cytokines like IL-6.

A system linked to RAS is the kallikrein-kinin-system (KKS) since ACE and ACE2 are regulators for degradation pathways within this system. The KKS is upregulated in inflammation and induces an increase in bradykinin via cleavage of kininogens by kallikrein (24). Bradykinin can bind to bradykinin2-receptor (B2R), which leads to vasodilatation and decreased blood pressure, thus serving as a natural counterbalancing system to RAS. In addition, bradykinin can be converted to des-Arg-BK (DABK), which binds and activates the bradykinin1-receptor (B1R). While B2R is constitutively expressed, the B1R is predominantly induced by inflammation, but constitutively expression was found in the central nervous system as well (25). As seen in bronchial alveolar lavage (BAL) of patients with COVID-19, B1R is upregulated and becomes highly expressed (26). DABK is the ligand to B1R and binding results in the stimulation of chemokines (CXCL), iNOS, and other inflammatory molecules (27). DABK can be degraded by ACE2, resulting in less activation of the inflammatory B1R pathway.

**Figure 3:**
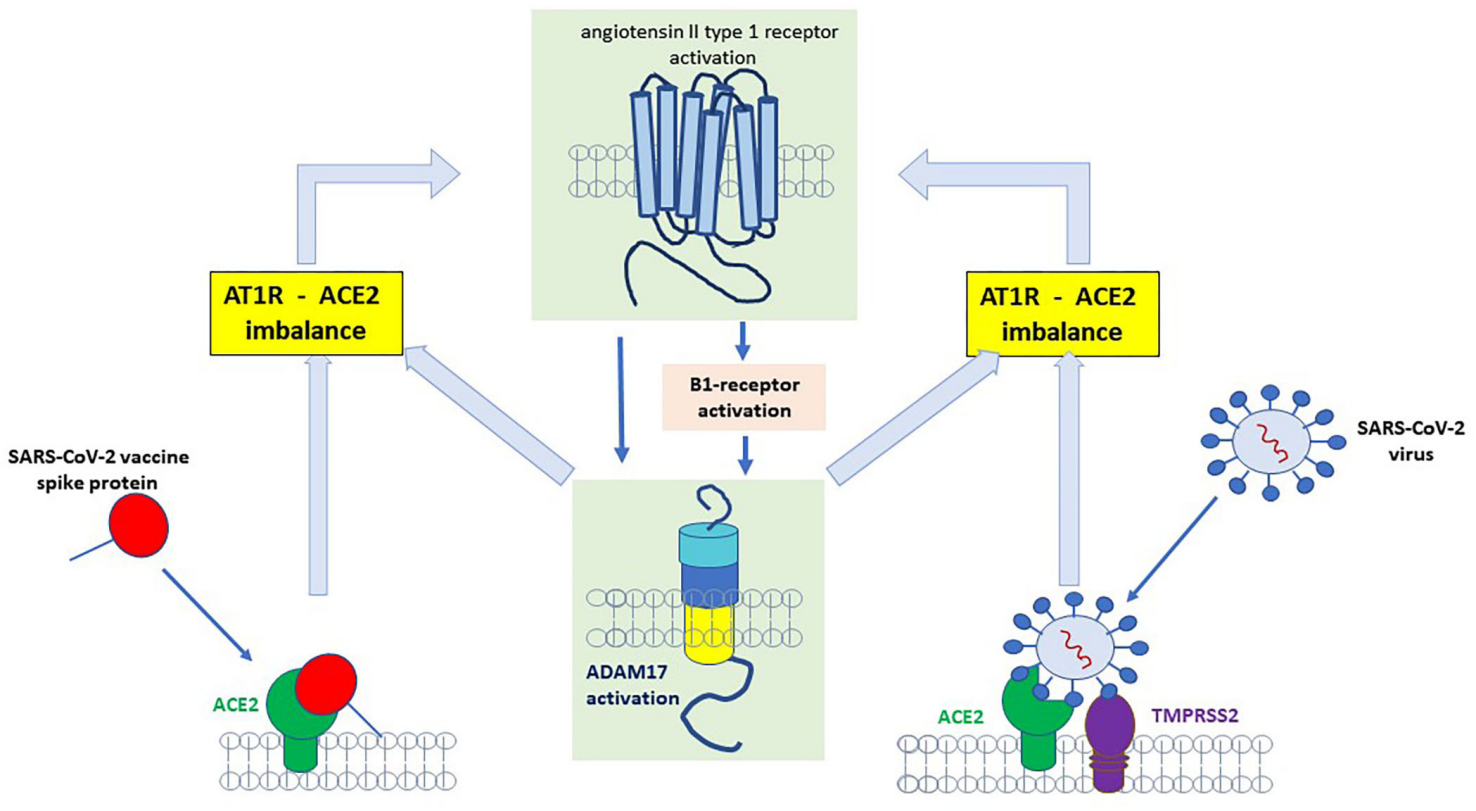
Binding of SARS-CoV-2 spike protein to angiotensin-converting enzyme 2 (ACE2) receptor results in the upregulation/activation of angiotensin II type 1 receptor (AT1)- and bradykinin B1 receptor (B1)-receptor which further induces a disintegrin and metalloprotease (ADAM)17 upregulation. ADAM17 is a sheddase for ACE2 and an increase in ADAM17 results in more degradation of ACE2. Less ACE2 will increase B1R (because degradation of [des-Arg9]-bradykinin (DABK) by ACE2 will decrease and more DABK is available for binding to B1R).

In this regard, Parekh reported that B1R activation induced a disintegrin and metalloprotease (ADAM) 17 upregulation in neurons (28). Since ADAM17 is mediating ACE2 shedding, this will result in less inactivation of DABK, which then can stimulate the B1R even more. In this case, it is tempting to speculate, that the inflammation induces a vicious circle via the B1R pathway (Figure 3). Another ADAM17-inducing pathway is the stimulation of AT1R (29). The upregulation of ADAM17 via AT1R can induce ACE2 shedding, which would then result in enhanced stimulation of the B1R and thus perpetuate the inflammatory B1R-driven response (29). A recent study by Pedrosa et al. showed *in vitro*, that the spike protein increases ADAM17 and reduces membrane-bound ACE2 (30). Thus, ADAM17 might not only be a key player in the regulation of RAS pathways but also elicits a critical role in the pathophysiology of COVID-19 and potentially in PVS as summarized in Figure 3.

As stated above, the shedding of ACE2 or fusion of the ACE2-spike protein complex results in enhanced stimulation of the B1R and perpetuates B1R driven inflammatory response (Figure 3) (29). The activation of B1R has been implicated in a variety of symptoms and diseases involving cardiovascular, neurological, dermatological, gastrointestinal, and autoimmunity (14). The B1R signaling cascade is involved in the regulation of nociception (31). Interestingly, not only nociception but also memory deficiency and cardiovascular disorders were restored in an amyloid mice model of Alzheimer's disease when the B1R was blocked (32). The underlying pathophysiology involves an increase in the blood-brain barrier which has been linked to the tissue kallikrein system and an imbalance equilibrium between B1R and B2R (summarized in Figure 2) (33). The activation of B1R results in increased vascular permeability, upregulation of iNOS, and prolonged production of NO (34). The wide expression of this mechanism might be one important pathway underlying the clinical variety of symptoms and organs involved. The gastrointestinal tract is emerging as one affected organ since fecal SARS-CoV-2 RNA has been found in 4% of patients 7 months after COVID-19 and was associated with gastrointestinal symptoms (35). Since intestinal epithelial cells express ACE2 and ADAM17 (36), rebalancing the intestinal RAS could be one important site as well. In summary, a dynamic imbalance within the RAS and subsequently a B1R-B2R disequilibrium may account for the symphony of symptoms seen in long-Covid patients and may represent a target for future drug therapy.

### Role of metalloprotease ADAM17 and its regulator TIMP3

Another enzymatic imbalance (integrated into the RAS signaling cascade) has been studied in COVID-19 patients which is the balance between the metalloproteinase ADAM17 and its endogenous inhibitor tissue inhibitor of metalloproteinase 3 (TIMP3). Both enzymes are critical for the maintenance of physiological processes in vascular cell repair, neurological disorders, and cancer growth (37). Increased ADAM17 level has been reported in alveolar epithelial cells after SARS-CoV-2 infection (36). as well as a tendency toward a decrease of its inhibitor TIMP3. Thus, it is very likely that infection or chronic inflammation as reported in long-Covid results in a persistent imbalance within the ADAM17/TIMP3 system. Moreover, it is worth speculating that the normalization of the RAS balance might subsequently contribute to a normalization of the ADAM17/TIMP3 balance as well. In general, controlling inflammatory and remodeling processes following injury or infection is a critical target for the restoration of physiology.

### Regulation mechanisms of central nervous RAS

Of special interest is the interaction of RAS within the central nervous system (CNS). AT1Rs are located in the brain (particularly in the putamen) and ANG II can increase the activity of excitatory neurons, especially within the paraventricular nucleus (PVN) and the putamen, resulting in chronic increased sympathetic hyperactivity (38). ACE2 is expressed within the PVN and plays a role in maintaining an inhibitory input involving the inhibitory GABA receptors. Thus, central RAS is involved in neurogenic hypertension. Evidence was provided by Mukerjee et al. (39) that ACE2 opposes autonomic dysfunction by sustaining inhibitory inputs to pre-sympathetic PVN neurons. In ADAM17-knockdown, the Ang-II induced blood pressure increase was blunted and neuronal activation of pre-sympathetic PVN neurons was reduced. In rats injection of IL-1β induced an increase in blood pressure and heart rate (40). In addition, an increase in central RAS activity in the subfornical organ (SFO) and the PVN was detected. This expression could be attenuated by local pretreatment with AT1R antagonist Losartan, ACE-inhibitor, or COX2-inhibitor. Thus, the impact of central RAS on autonomic function is likely to impact clinical symptoms in PASC and PVS as seen in postural-orthostatic-tachycardia syndrome (POTS) (41). Women are more often affected with PASC and PVS. Whether this is due to differences in central RAS regulation (42) in central RAS regulation or hormones remains to be elucidated.

Bali et al. (43) summarizes the involvement of RAS in pain, anxiety as well as learning and memory capacity. These symptoms appear regularly in patients with PASC and PVS. Moreover, there is evidence for a dual role of ANGII in nociception with both, increasing and decreasing pain perception in different settings and concentrations, which makes the understanding of RAS even more challenging (43). Nevertheless, these observations underline the importance of a balanced renin-angiotensin and kinin-system for the recovery of patients with PASC and PVS.

### Impact of a dysbalanced RAS on coagulation cascades

Following COVID-19 infection and SARS-CoV-2 vaccination, an increased risk of thrombotic events like pulmonary embolism, myocardial infarction, and stroke are reported (41, 44–46). Cytokine storm and hypoxia resulting in endothelial damage, platelet activation as well as activation of the coagulation cascade are discussed as the underlying mechanisms leading to coagulation abnormalities (47–49). Amyloid fibrin micro clots are detected in acute infection long-Covid and PVS suggesting microvascular complications as one part of the disease process (50). Even though there is evidence for a favorable outcome with early anticoagulation (49) data regarding extended post-discharge anticoagulation are more contradictory, thus updated guidelines should be followed (51). Since the kallikrein-kinin system can activate the coagulation system via factor XII (52) therapeutical interventions rebalancing RAS and the closely linked kallikrein-kinin system, can potentially decrease the hyperinflammatory and procoagulant status of long-Covid and PVS patients as well. Therefore, targeting RAS in long-Covid and PVS might be beneficial since blocking the AT1R restores RAS balance due to (1) a decrease of ADAM17, (2) an increase of ACE2 with subsequently, and (3) downregulation of B1R.

In contrast to canonical cardiovascular diseases, we are in favor of AT1R-antagonists (AT1RB) in long-Covid /PVS patients since we know for decades that ACE-inhibitors impair the ACE-dependent degradation of bradykinin. AT1RB were extensively studied *in-vitro* and in animal models and are in broad clinical use for decades with good clinical tolerability (53). They have an excellent safety profile but differ regarding their half-life as well as binding affinity and lipophilic entities. Moreover, as reported 20 years ago by our group (53), AT1RB can be adjusted by computational design to a higher binding affinity to other components of the renin-angiotensin system with similar protein binding sequences for example ACE2. In this regard, Ridgway and colleagues showed recently also using computational modeling that AT1RB can be modified toward a higher binding affinity to the ACE2-receptor (thereby competing/scavenging with SARS-CoV-2's spike protein) (54).

Candesartan and telmisartan were used in pilot trials (53). Particularly Telmisartan has a long half-life, high-affinity binding to AT1R as well as PPARγ modulating capacity, thereby eliciting an anti-inflammatory effect. Telmisartan treatment in patients with acute COVID-19 resulted in a more favorable outcome (55). In a beta-amyloid oligomer-induced microglia activation, telmisartan inhibited the induced inflammatory response and increased the anti-inflammatory IL-10 expression, pathways mediated by PPARγ (56). Nevertheless, whether differences in AT1RB could have an impact on the treatment of long-COVID and PVS should be evaluated in larger clinical trials.

### Lipid-metabolism and spike protein elimination

Cholesterol fractions, particularly those from low- and high-density lipoproteins are known to elicit multiple biological effects beyond cardiovascular diseases (57, 58). Inflammatory mediators are known to modify LDL/VLDL or HDL cholesterol fractions either by compositional changes, post-translational modifications of proteins, or alterations of lipids and other cargo molecules like apolipoproteins (59). Recent evidence occurred that cholesterol-rich lipid rafts serve as a platform for SARS-CoV-2 entry in which receptors or enzymes like ACE2 and others i.e., toll-like receptors (TLRs), transmembrane serine proteases (TMPRSS) are recruited for their interaction with the viral spike protein (60, 61). Treatment of pro-inflammatory lipid-vesicles is established in the therapy for hypercholesterolemia and reduce the risk of morbidity and mortality in cardiovascular diseases for decades (57). The drugs named “statins” inhibit the hydroxyl-methylglutaryl coenzyme A (HMG-CoA) reductase, the rate-limiting enzyme in cholesterol synthesis. Moreover, for the past decade statins were used not only to reduce cholesterol levels but for their pleitropic effects including improvement of endothelial function, anti-inflammation, and modulation of immune response thereby restoring the vascular redox balance in cardiovascular diseases and other diseases (57, 62). Recent reports from patients with severe COVID-19 disease showed an improved outcome on statin therapy (63, 64). In a mouse model simvastatin downregulated inflammation of SARS-CoV-2 induced pulmonary infection and reduced inflammatory mediators like IL-6, CCL5/Rantes, and TNFα, whereas ACE2 expression was upregulated (65). In addition, simvastatin blunted the bradykinin-induced permeability in an *in vitro* model of post-capillary venules (66). This is of particular interest since inflammation via the B1R pathway is likely to be one of the major stimuli following SARS-CoV-2 infection or the vaccine-induced imbalance of the RAS-bradykinin axis.

Endothelial dysfunction has been reported in patients with PASC (67). If the vaccines induce immunoglobulin-like anti-idiotype antibodies or other molecules that bind to ACE2 and induce a RAS imbalance, this would also result in endothelial dysfunction. Since statin therapy can improve endothelial dysfunction, this therapy might improve symptoms in PASC and PVS patients. Moreover, the anti-inflammatory and immune-modulating effects of statins are extensively reported previously (57, 68). They are widely used, accessible, affordable and show good safety profiles. For rebalancing the system, they are supposed to be used until the symptoms disappear which is only for 2–3 months which further decreases the risk of side effects. Whether statins can improve the clinical outcome in PASC and PVS by enhancing the reversed cholesterol efflux or preventing detrimental inflammatory effects promoted by pro-inflammatory cholesterol fractions requires future analysis.

### Dietary effects targeting mast cell activity

The gastrointestinal tract has not only nutritional function, but it also serves as a major regulatory factor of immune response and has an impact on the brain via the enteric-neuronal-brain system (69). Patients with PASC and PVS report a variety of symptoms that overlap with symptoms described in mast-cell-activation-syndrome (MCAS), namely urticaria, rhinitis, fatigue, dyspnea, dizziness, brain fog, abdominal pain, diarrhea, etc (70). Research within the last years has shown, that post-infectious irritable bowel syndrome can occur after infection and clinical symptoms might persist for many month (71). The underlying mechanisms involve low-grad-inflammation and MCAS. Recent data suggest that MCAS is associated with PASC (72). It has been shown before, that local intestinal reactions to food antigens (i.e., gluten, wheat, soy, milk) can result in local edema, mast cell activation, and abdominal pain (73) and there is evidence, that mast cells (MC) released histamine can not only activate visceral afferent and dorsal root ganglia but also sensitize them suggesting an increased reaction after stimulation (74).

A recent study reported that spike-mediated ACE2 internalization to endothelial cells was potentiated in the presence of histamine (75). Thus it is likely, that MC activation and the induced histamine release are involved in clinical symptoms, reported from patients with PASC and PVS.

SARS-CoV-2 can infect gastrointestinal tissue since enterocytes of the small intestine express ACE2 receptors (76). It is unlikely, that vaccination has an impact on intestinal-lumen ACE2 receptors directly, but vascular endothelial cells might be affected, resulting in RAS imbalance, local inflammation, and leaky gut. The importance of a healthy gut and its implication for the microbiome, the immune system, and the gut-brain axis has been extensively researched over the last years (69). Mast cells can be activated due to a variety of ligands to different receptors and release molecules like histamine, tryptase, chymase, serotonin, TNFα, kinins, and others (70). Since chymase can serve as an alternative pathway for the conversion of Ang-1-12 to Ang II, this could stimulate AT1R, especially when ACE2 is downregulated, thus pushing the imbalance of RAS, additionally. Allergy or helminth-induced IgE-mediated activation of MC might accelerate symptoms in predisposed patients. Therefore, detailed history taking should be considered about pre-existing allergies, parasite infections, or auto-immune predispositions. In case of preexisting known food intolerance, it might be helpful to avoid those nutrients as well. Due to clinical overlap in symptoms and a potential overlap in pathophysiological pathways, MC activation is likely to occur and histamine release from MC might represent a trigger of clinical symptoms in PASC and PVS. Thus, a low-histamine diet, as well as avoidance of already known food allergens, should be considered as well as histamine-receptor blockers (77) to improve patients' clinical symptoms. Patients often struggle with those changes in diet. Therefore, referring to a dietician is recommended. Moreover, APP-based monitoring and information might help to support patients' acceptance, compliance, and self-education.

### The cardiologist's view

**Figure 4:**
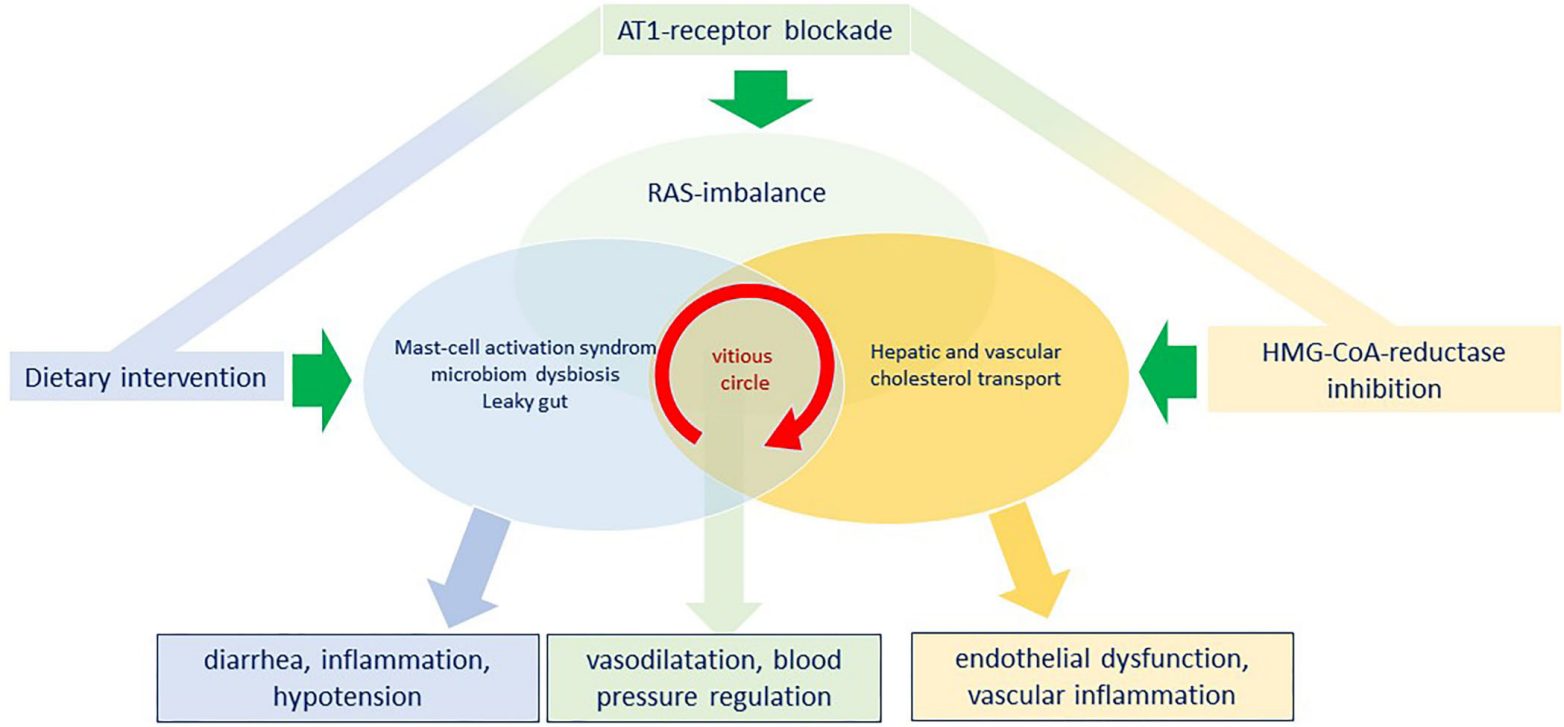
Model for a simultaneous triple intervention with angiotensin II type 1 receptor blockade, HMG-CoA-reductase inhibition and dietary intervention to penetrate the vicious circle of the vascular interplay between a renin-angiotensin-system (RAS)-imbalance, a continuous mast-cell activation with a microbiome dysbiosis and to reconstitute vascular function and reduce inflammation.

In an attempt to summarize the complex pathophysiology resulting from a dysbalanced RAS system, we would first highlight that long-Covid symptoms may occur either following SARS-CoV-2 infection and/or post-vaccination. The underlying mechanisms however require further analysis, but recent evidence supports a complex immune-inflammatory signaling network involving an imbalance within the RAS system as well as a B1R-B2R disequilibrium (Figure 2). It is therefore unlikely that a therapeutic approach targeting just one single step in a complex system will be sufficient and effective enough to treat such a complex syndrome. Therefore, we here propagate a multidimensional approach targeting major regulating pathways involved namely the RAS-bradykinin axis, the cholesterol metabolism, and finally the intestinal microbiome (Figure 4). In detail, AT1RB rebalances the RAS-bradykinin-axis, statin-therapy improves endothelial function and elicits pleiotropic anti-inflammatory effects all together stimulating immunomodulatory pathways. These two drug therapies should be governed by a histamine-reduced diet limiting mast-cell effects, reducing inflammation, and potentially restoring the intestine microbiome. Lifestyle changes like a histamine-reduced diet are challenging for patients, particularly in the light of pre-existing or newly developing food intolerances or allergies. Thus, referral to a dietician should be considered. Nowadays APPs and digital information from institutional websites can support patients' acceptance, compliance, and self-education.

So far, no biomarker has been evaluated for PASC and PVS. The variety of symptoms makes it necessary to evaluate patients carefully and individually to exclude other causes or comorbidities, especially microclots or reactivation of other infections, which have been reported for PASC and PVS (78, 79). The variety of symptoms in PASC and PVS with the need for extensive evaluation and workup is challenging for patients and clinicians and aggravates the design of clinical trials. Since there is an urgent need for therapies that improve clinical outcomes which are widely applicable and with low risk for side effects, we proposed the following strategy: A combined treatment using statins plus angiotensin II type 1 receptor antagonist on the base of a histamine reduced diet. Rebalancing the SARS-CoV-2 or vaccine-induced disturbances with a multidimensional approach might be a promising way to treat patients with PASC and PVS and should be evaluated. It is not the end of the long-COVID story but it might extend our armamentarium to protect patients from detrimental spike-protein effects. Detailed genetic and molecular studies, as well as translational and randomized clinical trials, are warranted to evaluate this first treatment algorithm.

## Data availability statement

The original contributions presented in the study are included in the article/supplementary material, further inquiries can be directed to the corresponding author/s.

## Author contributions

Both authors listed have made a substantial, direct, and intellectual contribution to the work and approved it for publication.

## Funding

This work was supported by the Dr. Reinfried Pohl Foundation. Open Access funding was granted by the Open Access Publication Fund of the Philipps-Universität Marburg supported by the Deutsche Forschungsgemeinschaft (DFG, German Research Foundation).

## Conflict of interest

The authors declare that the research was conducted in the absence of any commercial or financial relationships that could be construed as a potential conflict of interest.

## Publisher's note

All claims expressed in this article are solely those of the authors and do not necessarily represent those of their affiliated organizations, or those of the publisher, the editors and the reviewers. Any product that may be evaluated in this article, or claim that may be made by its manufacturer, is not guaranteed or endorsed by the publisher.

## Abbreviations

post-VAC: post vaccination
Covid-19: coronavirus disease 2019
RAS: renin-angiotensin system
B1R: bradykinin1 receptor
statins: HMG-CoA reductase inhibitors
PASC: post-acute sequelae COVID-19
DABK: [des-Arg9]-bradykinin
ADAM17: A disintegrin and metalloprotease 17
ACE: angiotensin converting enzyme
AT1R: angiotensin II type 1 receptor
AT1RB: angiotensin II type 1 receptor antagonist/blockade.
