# Corrigendum: The rationale for the treatment of long-COVID esymptoms—a cardiologist's view

**DOI:** 10.3389/fcvm.2023.1244340

**Published:** 2023-07-21

**Authors:** Elisabeth Schieffer, Bernhard Schieffer

**Affiliations:** Department of Cardiology, Angiology and Critical Care Medicine, University Hospital Marburg (UKGM), Philipps University Marburg, Marburg, Germany

**Keywords:** lipid – hybrid nanoparticles, HDL - cholesterol, long-Covid syndrome, histamin challenge, cholesterol, renin-angiotensin system, kalkrein-kinin system

A Corrigendum on The rationale for the treatment of long-COVID symptoms—a cardiologist’s view By Schieffer E and Schieffer B. (2022) Front Cardiovasc Med. 9:992686. doi: 10.3389/fcvm.2022.992686

In the published article, there was an error in the article type. It was incorrectly published as a Systematic Review. The correct article type is: Perspective.

The authors apologize for this error and state that this does not change the scientific conclusions of the article in any way. The original article has been updated.

